# Effects of Date Palm Waste Compost Application on Root Proteome Changes of Barley (*Hordeum vulgare* L.)

**DOI:** 10.3390/plants12030526

**Published:** 2023-01-23

**Authors:** Emna Ghouili, Khaled Sassi, Yassine Hidri, Hatem Cheikh M’Hamed, Anil Somenahally, Qingwu Xue, Moez Jebara, Rim Nefissi Ouertani, Jouhaina Riahi, Ana Caroline de Oliveira, Ghassen Abid, Yordan Muhovski

**Affiliations:** 1 Laboratory of Legumes and Sustainable Agrosystems, Centre of Biotechnology of Borj-Cedria, (L2AD, CBBC), P.O. Box 901, Hammam-Lif 2050, Tunisia; 2 Laboratory of Agronomy, National Agronomy Institute of Tunisia (INAT), University of Carthage, Avenue Charles Nicolle, Tunis-Mahrajène, P.O. Box 43, Tunis 1082, Tunisia; 3 Laboratory of Integrated Olive Production in the Humid, Sub-humid and Semi-arid Region (LR16IO3), Olive Tree Institute, Cité Mahragène, P.O. Box 208, Tunis 1082, Tunisia; 4 Agronomy Laboratory, National Institute of Agronomic Research of Tunis (INRAT), Carthage University, Hedi Karray Street, Ariana 2049, Tunisia; 5 Department of Soil and Crop Sciences, Texas A&M University, 370 Olsen Blvd, College Station, TX 77843-2474, USA; 6 Texas A&M AgriLife Research and Extension Center, Amarillo, TX 79403-6603, USA; 7 Laboratory of Plant Molecular Physiology, Centre of Biotechnology of Borj Cedria, P.O. Box 901, Hammam-Lif 2050, Tunisia; 8 Biological Engineering Unit, Department of Life Sciences, Walloon Agricultural Research Centre, Chaussée de Charleroi, P.O. Box 234, 5030 Gembloux, Belgium

**Keywords:** barley, compost, differentially abundant proteins, proteome, qRT-PCR, roots

## Abstract

Proteomic analysis was performed to investigate the differentially abundant proteins (DAPs) in barley roots during the tillering stage. Bioinformatic tools were used to interpret the biological function, the pathway analysis and the visualisation of the network amongst the identified proteins. A total of 72 DAPs (33 upregulated and 39 downregulated) among a total of 2580 proteins were identified in response to compost treatment, suggesting multiple pathways of primary and secondary metabolism, such as carbohydrates and energy metabolism, phenylpropanoid pathway, glycolysis pathway, protein synthesis and degradation, redox homeostasis, RNA processing, stress response, cytoskeleton organisation, and phytohormone metabolic pathways. The expression of DAPs was further validated by qRT-PCR. The effects on barley plant development, such as the promotion of root growth and biomass increase, were associated with a change in energy metabolism and protein synthesis. The activation of enzymes involved in redox homeostasis and the regulation of stress response proteins suggest a protective effect of compost, consequently improving barley growth and stress acclimation through the reduction of the environmental impact of productive agriculture. Overall, these results may facilitate a better understanding of the molecular mechanism of compost-promoted plant growth and provide valuable information for the identification of critical genes/proteins in barley as potential targets of compost.

## 1. Introduction

Barley (*Hordeum vulgare* L.) is one of the world’s major cereal crops with high protein content and ranked fourth, after wheat, corn and rice. In temperate countries, such as Tunisia, barley is commonly used for human food, animal feed and malt production. In comparison with other cereal crops, barley is better suited to dry conditions and is usually grown in semi-arid and arid regions. Moreover, barley is the most salt-tolerant cereal plant and is considered to be a moderately salt-tolerant crop [1]. Low soil fertility and poor agronomic practices are among the major constraints responsible for the low productivity of barley in southern areas of Tunisia. Indeed, the average barley grain yield was 0.85 t ha^−1^ lower than that of worldwide productivity (3.25 t ha^−1^) [2]; yields are characterised by their instability and thus vary significantly from year to year. The use of organic amendments such as compost has been shown to improve soil quality and fertility, promoting plant growth and increasing crop productivity [3].

The application of compost increased soil microbial biomass, increased total soil carbon and nitrogen, resulting in improved radish (*Raphanus raphanistrum* subsp. *sativus* ‘Cherry Belle’, Burpee) growth and development [4]. Similar results were reported in tomato [5] and roselle [6] plants. Compost positively affects root growth and development of grapevine [7] and a beneficial effect on soil fertility, yield, and grape quality was also recorded. A significant increase in the germination rate of green bean seeds was observed upon the addition of different concentrations of composts derived from municipal solid waste, suggesting that compost consistently promotes biological activity, which can increase germination [8]. Under drought stress conditions, several studies have reported positive effects of compost incorporation to increase the water-retention capacity of soil, which may lead to an increase in growth and yield in crops such as canola [9] and wheat [10]. In this context, the application of date palm waste compost at 30 t ha^−1^ induces the expression of nutrient transporter genes in roots of barley, which leads to improved macronutrient uptake by the plant and then promotes plant growth, plant development, yield and yield components of barley grains [11]. Furthermore, the use of compost in agriculture stimulates plant immunity against pathogens, leading to a higher crop yield. Indeed, the application of compost improves the yield and the quality of tomatoes by suppressing vascular wilt caused by *Verticillium dahlia* [12]. Compost applications may be an effective strategy for promoting plant growth and development through various physiological events and thereby improving agricultural productivity; however, the modes of action describing the molecular mechanisms underlying the compost effects on plants are still largely unknown.

Recently, omics approaches, such as transcriptomic, proteomic and metabolomic, have contributed to the characterisation of the molecular mechanism of action of some biostimulants, as well as identifying their regulatory role in molecular and biochemical pathways [13]. Several studies have successfully used the power of proteomics as a discovery tool to uncover the mode of action of biostimulants and biofertilizers on plants at cellular levels [14]. Therefore, understanding the subcellular localization and post-translational modifications of proteins is crucial for a comprehensive understanding of plant responses to organic amendments, biostimulants and biofertiliser application. Recently, mass spectrometry-based proteomics has emerged as a broadly effective means for deciphering complex proteomic networks and provides a holistic view at the molecular level that may better reflect the phenotype of plant [15]. In this context, proteomics, as multidisciplinary omics sciences, provides key fundamental knowledge on the mode of action of compost on crop plants including barley. Unfortunately, no emerging studies in the literature that investigate the potential and effectiveness of proteomics approaches reveal the modes and mechanisms of the action of compost at molecular levels.

Proteome analysis of the barley malt rootlet proteome suggested the upregulation of secondary metabolism, reactive oxygen species (ROS) detoxification and protein biosynthesis pathways coordinated by phytohormones, especially jasmonic acid and auxin [16]. Furthermore, during barley seed germination, proteomic analysis revealed that the upregulation of proteins is mainly related to genetic information processing and carbohydrate metabolism pathways [17]. Nevertheless, no information is available on the effects of compost application on proteome, transcriptome and metabolome changes on barley. The objective of the present study was to understand the physiological mechanisms underlying barley plant responses to date palm waste compost. Hence, LC-MS/MS analysis was applied to evaluate the changes in the proteome of barley roots following compost treatment. To the best of our knowledge, this is the first comprehensive investigation to characterise the potential functional proteins involved in barley plant growth and the development process induced by date palm waste compost.

## 2. Results

### 2.1. Effect of Date Palm Waste Compost on Growth Parameters and Yield of Barley Crop

**Figure 1:**
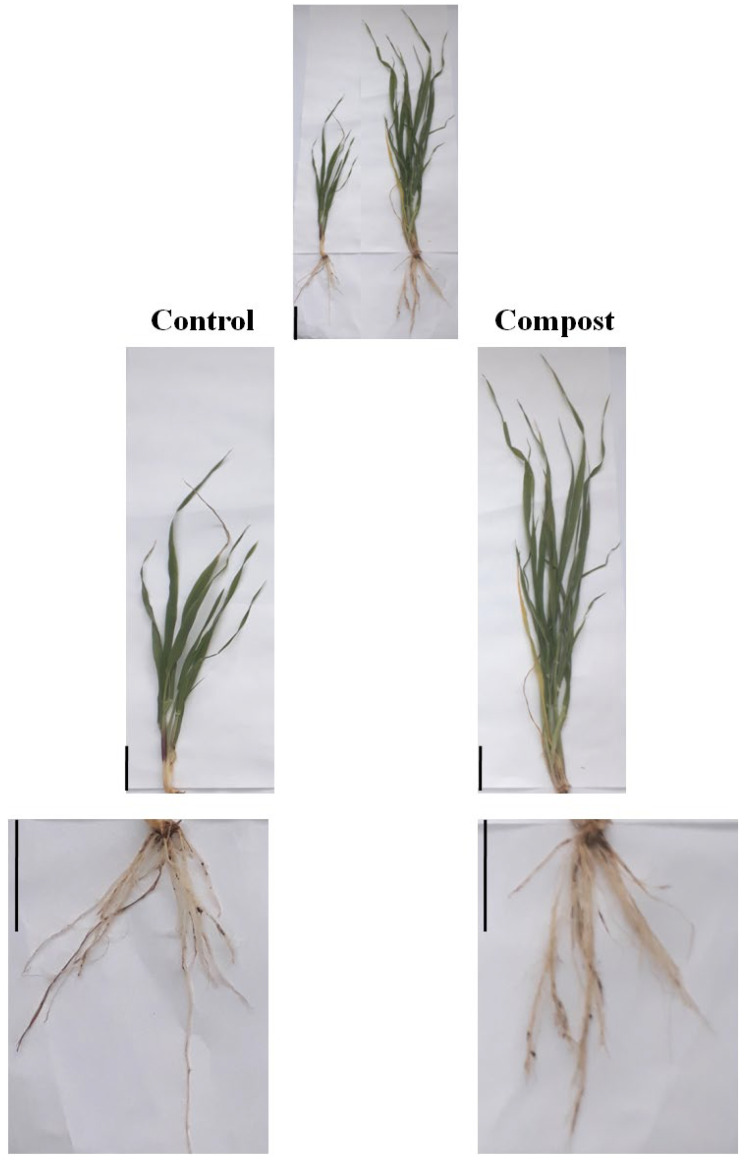
Morphological effects of date palm waste compost in shoots and roots of barley plants. Scale bars: 5 cm.

The use of organic fertilizer showed a significant effect on the growth of barley seedlings at the tillering stage (Figure 1).

The application of date palm waste compost at the rate of 30 t ha^−1^ significantly increased the plant height, shoot and root dry weight compared with control treatment (Table 1). Indeed, statistical analysis regarding plant height at the tillering stage showed significant differences for plots treated with date palm waste compost compared with the control. Compost-treated plants produced a greater plant height which was 49.66 ± 1.52 cm, representing a 55.18% increase over the control. A similar effect was noticed on the shoot and the root dry weight, which increased by 115.46% and 22.03%, respectively. However, the addition of date palm waste compost did not produce any significant effect on the root length of barley plants. Interestingly, statistical data of grain yield revealed that it is positively increased by compost addition. Grain yield was significantly increased by 49.82%. The greater value was observed in the compost treatment (4.33 ± 0.67 t ha^−1^), while the lowest value was observed in the control treatment (2.89 ± 0.54 t ha^−1^).

### 2.2. Identification of DAPs Using LC-MS/MS

In order to investigate the influence of date palm waste compost on proteome changes in barley roots, mass spectrometry analysis of compost-treated samples of barley roots was performed and resulted in the identification of 2580 non-redundant protein groups (Supplemental Table S1). To obtain DAPs, one-to-one comparison between the control and the compost-treated plants classified 72 proteins as DAPs. Statistical analysis showed that DAPs were considered as significantly altered (*p*-value < 0.01) with a fold change of ≥1.5 (upregulated proteins) or with a fold change of ≤1/1.5 (downregulated proteins). A total of 33 proteins were identified as upregulated and 39 proteins were downregulated (Table 2). Among the 33 upregulated identified proteins, three proteins showed increased levels of over four-fold compared with the control, three proteins showed increased levels of over three-fold compared with the control, six proteins showed increased levels of over two-fold compared with the control, and the remaining 21 proteins showed increased levels between one-point-five- and two-fold compared with the control. In the proteins with decreased expression, the variations of 20 downregulated proteins ranged from 0.66–0.5-fold compared with control, while the remaining 19 proteins among these changed by less than half-fold (Table 1).

### 2.3. Functional Categories and Subcellular Localisation of DAPs

**Figure 2:**
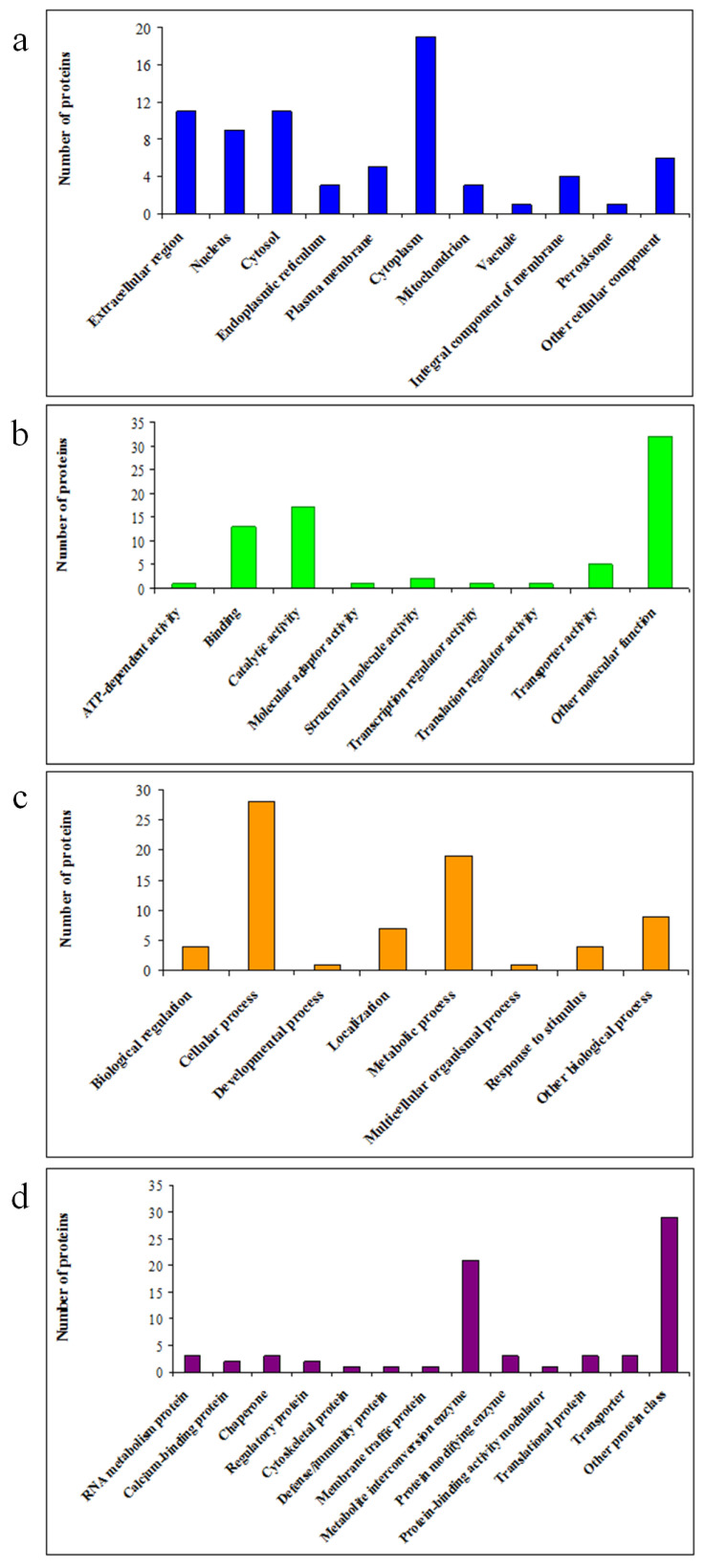
Functional categories and subcellular localization of DAPs from roots: subcellular localization (**a**), molecular function (**b**), biological process (**c**) and protein class (**d**).

The DAPs were subjected to Gene Ontology (GO) functional categories of biological process, molecular function, protein class and cell component in order to identify the influence of date palm waste compost on proteome changes in barley roots. The subcellular localisation analysis (Figure 2a) showed that these proteins were mainly localised in the cytoplasm (26%). In addition, 15%, 12.30%, 15%, 4.10%, 7%, 4.10%, 1.40%, 5.50%, 1.40% and 8.20% of the DAPs were localised in the extracellular region, nucleus, cytosol, endoplasmic reticulum, plasma membrane, mitochondrion, vacuole, integral component of membrane, peroxisome and other cellular components, respectively. Based on molecular function, these 72 identified DAPs were classified into nine categories (Figure 2b) including ATP-dependent activity (1.40%), binding (17.8%), catalytic activity (23.3%), molecular adaptor activity (1.40%), structural molecule activity (2.8%), transcription regulator activity (1.40%), transporter activity (7%), translation regulator activity (1.40%) and other molecular function (43.5%). According to GO analysis of biological processes (Figure 2c), DAPs were significantly enriched in the biological process of cellular process (38.30%), biological regulation (5.50%), developmental process (1.40%), localisation (9.60%), metabolic process (26%), multicellular organismal process (1.40%), response to stimulus (5.50%) and other biological processes (12.30%). In the protein class category (Figure 2d), metabolite interconversion enzyme (28.80%) was the most representative. Among these categories, a few DAPs were closely correlated with RNA metabolism protein (4.10%), calcium-binding protein (2.8%), chaperone (4.10%), regulatory protein (2.8%), cytoskeletal protein (1.40%), defense/immunity protein (1.40%), membrane traffic protein (1.40%), protein-modifying enzyme (4.10%), protein-binding activity modulator (1.40%), translational protein (4.10%), and transporter (4.10%).

### 2.4. KEGG Pathway Enrichment Analysis of DAPs Induced by Date Palm Waste Compost Application in Barley Roots

**Figure 3:**
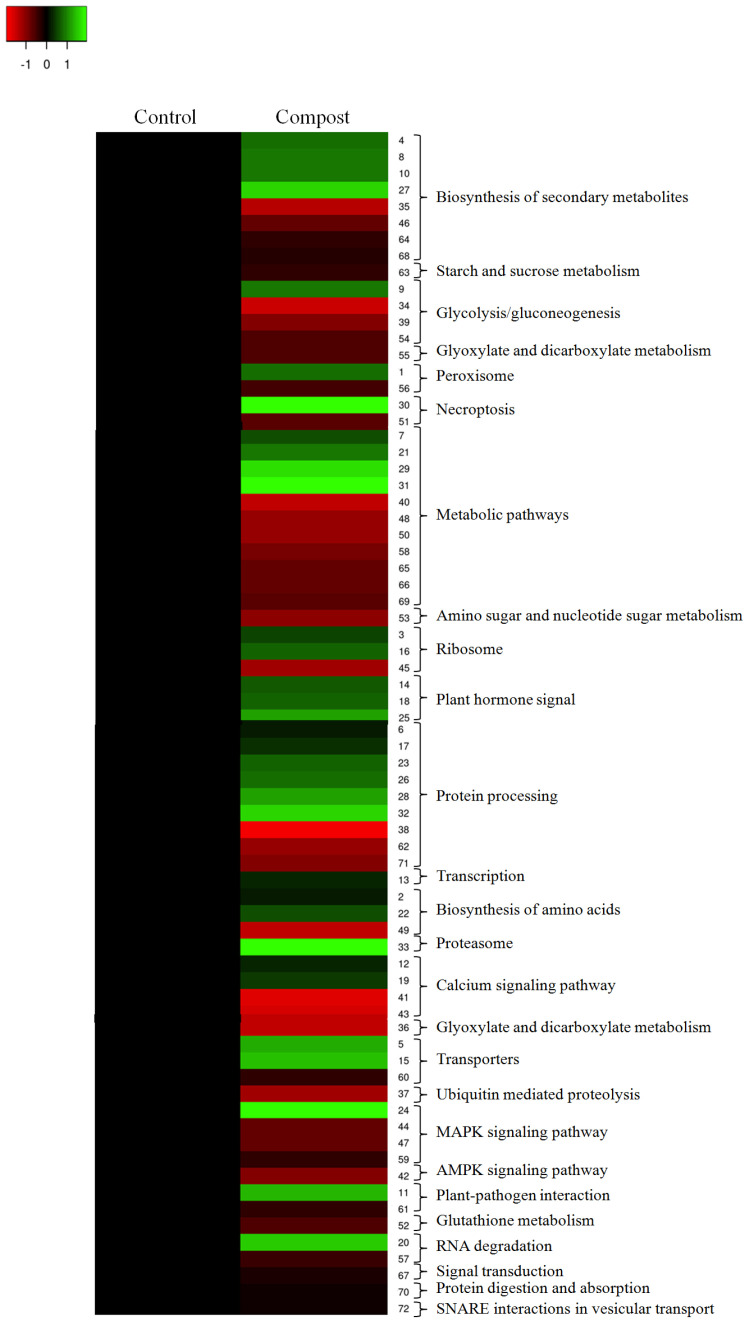
KEGG pathway enrichment of DAPs induced by date palm waste compost in barley roots.

To further understand the characteristics of DAPs, analyses of the Kyoto Encyclopedia of Genes and Genomes (KEGG) pathways was performed (Table 2). The results showed that the most significantly enriched pathways for root compost-treated plants included metabolic pathways, protein processing and biosynthesis of secondary metabolites (Figure 3). KEGG analysis also showed that the glycolysis/gluconeogenesis, the calcium signaling pathway and the MAPK signaling pathway were enriched. These diverse groups of proteins were enriched by both upregulated and downregulated proteins. All these results indicated that the different responses to compost application between control and treated plants were at proteomic levels.

### 2.5. Validation of the Expression of DAPs in Barley Roots by Quantitative Real-Time Polymerase Chain Reaction (qRT-PCR)

**Figure 4:**
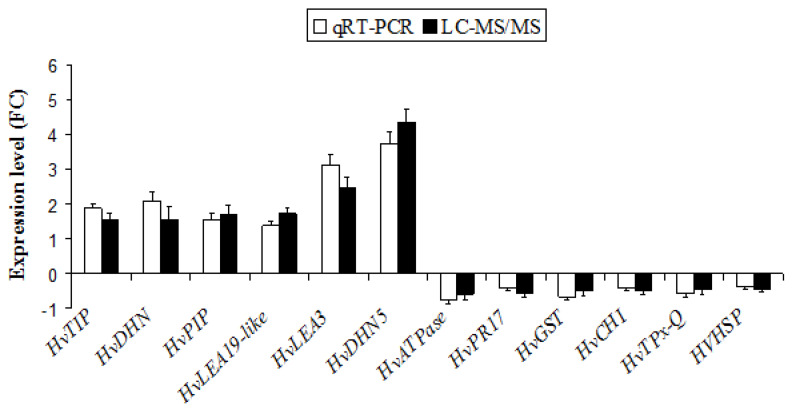
Confirmation of proteomic results by quantitative real-time PCR (qRT-PCR). The white and black bars represent fold change of mRNA and protein abundances in barley roots, respectively.

In order to validate the quality of the proteomics, qRT-PCR analysis was performed to confirm the correspondence between mRNA transcription and protein expression (Figure 4). Twelve genes encoding to *HvATPase*, *HvPR17*, *HvGST*, *HvCHI*, *HvTPx-Q*, *HVHSP*, *HVTIP*, *HvDHN*, *HvPIP*, *HvLEA19-like*, *HvLEA3* and *HvDHN5*, which were differentially expressed in the barley roots revealed by the proteomic analysis, were selected for quantification (Figure 4). Of these 12 proteins, six proteins (HvPIP, HvLEA19-like, HvDHN5, HvPR17, HvCHI and HVHSP) coincided well with their corresponding coding genes (Figure 4). However, the transcript expression levels of other genes differed slightly from the relative protein levels, which may be due to various post-translational modifications and post-translational modification under the compost application, such as protein phosphorylation and glycosylation. In general, the expression patterns of studied genes were consistent with LC-MS/MS proteomics data, suggesting that the results of qRT-PCR were consistent with those derived from the proteomics analysis and the quality of the proteomics data is acceptable. Moreover, to support the reliability of LC-MS/MS sequencing data, a correlation coefficient (R2) (of the fold changes between qRT-PCR and LC-MS/MS sequencing) of 94.89% was obtained (Supplemental Figure S1).

### 2.6. Putative Protein-Protein Interaction (PPI) Networks of DAPs to Date Palm Compost Waste Application

**Figure 5:**
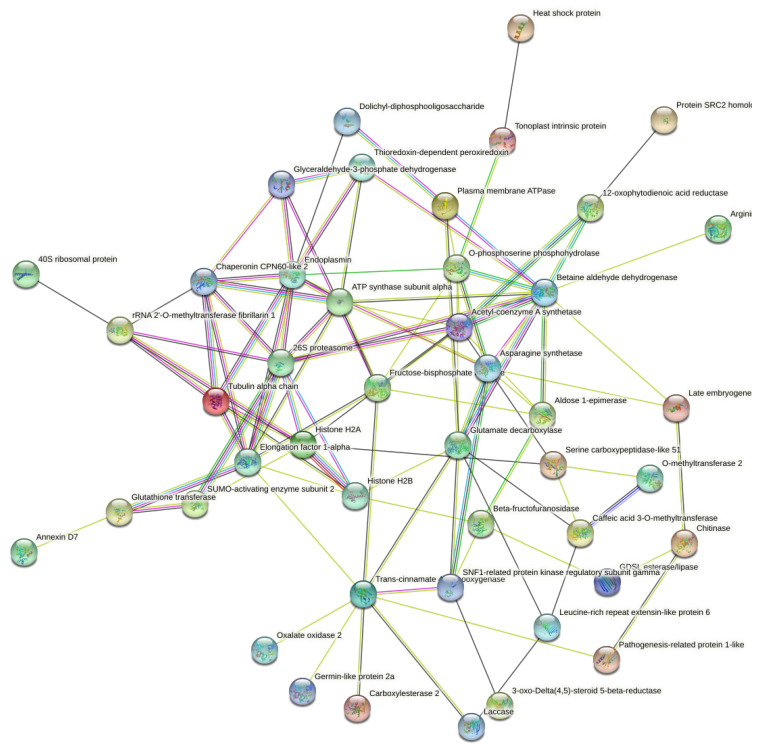
Analysis for protein-protein (PPI) interaction network for the DAPs. The different line colours represent the types of evidence used in predicting the associations: neighbourhood (green), co-occurrence across genomes (blue), co-expression (black), experimental (purple) and association in curated databases (light blue) or texting (yellow). Disconnected nodes or proteins not connected to the main network were hidden in the network.

To uncover the interaction networks for the DAPs identified in the root tissue of the barley plants treated with date palm waste compost, a protein-protein interaction (PPI) network was performed using a STRING database. Forty-five proteins out of the seventy-two DAPs identified in this work comprise the interaction network (Figure 5). This analysis demonstrated that the identified DAPs are highly interconnected. Hub analysis of the network suggested endoplasmin, betaine aldehyde dehydrogenase, trans-cinnamate 4-monooxygenase, caffeic acid 3-O-methyltransferase, and histone H2B were central proteins in these networks and in the barley plant’s response to the date palm waste compost application. These proteins were upregulated and play key roles in protein processing, metabolic pathways and biosynthesis of secondary metabolites.

## 3. Discussion

### 3.1. Date Palm Waste Compost Promote the Growth and Development of Barley Plants

In this study, the plant height, the shoot and root dry weight, and the grain yield of barley was increased significantly due to the application of date palm waste compost. Similar observations were reported with some amendments including compost [18]. As a consequence, growth and yield parameters of the barley plant such as the plant height, the fresh and dry weight were significantly improved with the organic amendments treatment. Furthermore, the increase in the growth of the compost-treated plants might be due to the presence of plant growth substances such as humic substances and plant growth hormones including auxins, gibberellins and cytokinins [19]. The increased shoot and root dry weight of the barley plant may increase the uptake of essential plant nutrients such as N, P and K, which play an important role in supporting plant growth and crop productivity through the maximum exploitation of soil. In addition, the compost application had a considerable effect on the yield compared with the untreated control. There was an observable increase in the yield of the barley treated with date palm waste compost. The compost significantly increased the grain yield and some yield components such as the number of spikes per m^2^ and the 1000-grain weight of barley [20]. In wheat (*Triticum aestivum*), applications of compost had a significant effect on the yield and yield components such as the number and the weight of grains per spike, as well as the macronutrient uptake [21]. Based on these findings, it may be recommended that the application of date palm waste compost at the rate of 30 t ha^−1^ is considered economical and suitable for growing barley.

### 3.2. Date Palm Waste Compost Induced Changes in the Barley Root Proteomes

The application of compost as an organic amendment was previously reported to stimulate root biomass in barley plants [22], to induce alterations in the root transcriptome [11] and to improve the barley tolerance to abiotic stress [18]. To our knowledge, no proteomics work has been reported to address the effect of date palm waste compost on the proteome of barley roots to understand the responses triggered by an organic amendment at the molecular level. In this study, the application of compost resulted in global changes in the proteome of barley roots. A total of 72 DAPs were responsive to 30 t ha^−1^ in root tissues, suggesting that the impact of compost at the molecular level correlated to stimulatory effects on plants such as an increase in root biomass.

### 3.3. Detoxification Proteins

Proteins related to redox homeostasis such as glutathione S-transferase (GST) and thioredoxin-dependent peroxiredoxin (TPx-Q) were identified in this study, whose abundance decreased in response to the date palm waste compost application. This finding suggests the possible role of compost in reducing the oxidative damage by reducing antioxidant enzymes activity and reducing the reactive oxygen species (ROS) generation in compost-treated barley plants. Similarly, other classes of biostimulants including humic substances (HS) and the plant growth-promoting strain *Pseudomonas putida* AKMP7 have been reported to reduce the expression of genes related to redox homeostasis such as GST, superoxide dismutase (SOD), ascorbate peroxidase (APX) and catalase (CAT) in Arabidopsis [23] and wheat [24]. Elevated ROS levels are harmful to plants and may alter various biological processes leading to plant death. Therefore, to combat oxidative damage due to ROS, plants have evolved a complex antioxidant system, including both enzymatic and non-enzymatic defenses. In this context, the compost used in this study originates from date palm wastes, which are rich in antioxidants including polyphenols stored by plants in their fruits and leaves [25], which if applied to a barley crop, may promote beneficial effects by reducing potential oxidative threats. Moreover, the decrease in GST and TPx-Q abundances in the roots of barley plants exposed to compost indicates that the date palm waste compost used in this study is not favourable in creating oxidative stress in barley plants. Therefore, this used compost probably contains compounds that are not potentially phytotoxic and not conducive to oxidative stress. Conversely, the abundance of several DAPs related to redox homeostasis including GST and thioredoxins (Trxs) was increased in maize roots treated with protein hydrolysate (PH)-based biostimulants [26]. 

### 3.4. Plant Stress and Defense Proteins

Interestingly, several stress-related and responsive DAPs were differentially regulated with date palm waste treatment consistent with the different organic amendments previously reported, such as biochar [27]. In addition to organic amendments, several classes of biostimulants have been demonstrated to induce stress-responsive and related mechanisms and to alleviate the adverse effects of biotic and abiotic stresses in crops [28]. This is not surprising; and in addition to improving the productivity of crops, composts are extensively applied to prevent the negative stress effects in plants [29]. Proteomics analyses showed that several important classes of stress responsive proteins including late embryogenesis abundant protein (LEA) and heat shock protein (HSP) were differentially expressed in response to the compost treatment. 

The compost treatment induced the abundance of abcissic acid stress and ripening (ASR), which is involved in ABA signalling. The upregulation of this gene is related to the increase of ABA activity, correlated to an increase in stress tolerance [30]. Some ASR proteins function as transcription factors involved in plant response to abiotic and biotic stresses and in several physiological processes such as fruit development and ripening [31]. Similarly, the expression of ABA related genes such as WRKY transcription factor, trehalose 6-phosphate phosphatase (T6PP), ABA 8′-hydroxylase and protein phosphatase 2C was increased in the root tissue of a tomato due to the application of a tannin-based biostimulant [32].

LEA3, LEA 19-like (a subgroup of LEA3), as well as two proteins encoding dehydrin belonging to the late embryogenesis abundant (LEA) proteins family that is involved in reducing cellular injuries and closely associated with resistances to abiotic stresses, especially to drought, were upregulated by compost treatment. Several studies reported that the enhanced expression of LEA proteins has led to increased resistance to dehydration, as well as other stresses such as heavy metals, heat, cold and salinity in many plant species [33]. Other studies have also found that the application of a biostimulant based on tannins increases the expression of two different LEA proteins [28]. ASR encoding of an LEA protein classified into the LEA7 group [34]. In this study, the compost primed the proteins involved in the response to abiotic stimuli and the upregulation may suggest a positive and protective effect of the compost application on the barley plants.

The compost application did significantly suppress plant diseases caused by different plant pathogens. Indeed, compost treatment effectively controls *Phytophthora capsici* Leonian (PHC) that affects summer squash (*Cucurbita pepo* var. *cylindrica* L.) [35]. Therefore, the expression patterns of DAPs involved in plant-pathogen interactions were characterised in the study. Four DAPs which were associated with pathogenesis such as pathogenesis-related protein 17 (PR17), pathogenesis-related protein 1-like, chitinase (CHI) and germin-like protein (GLP) were mostly downregulated under the compost treatment.

In addition, the compost treatment downexpressed the 12-oxophytodienoic acid reductase (OPR) in the barley root tissue. This protein is involved in the synthesis of jasmonic acid (JA), which is associated with plant defense against herbivory and necrotrophic pathogen attacks [36] and positively regulates plant immunity during hemibiotrophic pathogen infection [37]. Chitin elicitor binding protein (CEBiP) is another protein associated with the plant defense system, which was also downregulated by the compost treatment. CEBiP is involved in the perception of chitin oligosaccharides which act as pathogen-associated molecular patterns (PAMPs) or microbe-associated molecular patterns (MAMPs) that activate the plant defense system such as the accumulation of chitinases [38]. This shows that compost has a potential application as a fertilizer and its application produces a positive effect on the plants by alleviating biotic stresses caused by external pests and pathogens on barley plants. Overall, in this study, the date palm waste compost application downregulated the defensive systems of the barley plants, indicating that the cell wall biogenesis was related to a direct vegetative growth instead of a defensive mechanism. Moreover, these results also suggest that the compost treatment probably activated the plants’ defense mechanisms in an earlier stage and then the energy was spent on the barley plants’ growth and development.

### 3.5. Transport Proteins

The abundance of proteins related to the transport of water and other small neutral molecules across membranes, such as the plasma membrane intrinsic protein (PIP) and the tonoplast intrinsic proteins (TIP) subfamily of aquaporins (AQPs), increased in response to the compost treatment. These proteins aid with the movement of water and solutes at the molecular level, leading to the better absorption of nutrients and water efficiency, resulting in an increase in overall plant growth. Furthermore, an increase in aquaporin 1 (PIP1) gene expression was observed in the roots of maize seedlings treated with humic acids [32].

### 3.6. Carbohydrate and Energy Metabolism Proteins

A number of DAPs identified from the barley roots were found to be involved in plant energy metabolism. Some of these enzymes are related to carbohydrate metabolism including glycolysis, such as aldose 1-epimerase (AEP1), β-1,3-1,4-glucanase, glyceraldehyde-3-phosphate dehydrogenase (GAPDH), fructose-bisphosphate aldolase (FBPA), sucrose 1-fructosyltransferase (1-SST), SNF1-related protein kinase (SnRK1) and acetyl-coenzyme A synthetase (AcsA). All of these proteins were differentially expressed in the roots of the compost-treated plants. The levels of AEP1 were increased in the compost-treated plants. Interestingly, AEP1 is a key enzyme of carbohydrate metabolism, catalysing the interconversion of the α- and β-anomers of hexose sugars such as glucose and galactose, which could be used as a crucial source of energy [39]. These results suggest that applying compost might enhance the flow of carbon through sugar metabolism, which accelerates the carbon assimilation pathway, consequently improving barley growth and stress acclimation. The decrease in the abundance of the laccase (LAC) protein further supports the idea that sufficient carbohydrates were available for the compost-treated plants. This is based on the evidence that laccase genes are typically only upregulated when carbohydrates are not readily available and lignin degradation becomes necessary, or during high phenolic stress. The upregulation of β-1,3-1,4-glucanase that degrades mixed linkage glucan, a transient wall polysaccharide found in cereals such as barley, may lead to the increased energy production required for rapid seedling growth [40]. These results were consistent with several previous investigations [23,26,41], who reported that protein hydrolysate, glutamine and humic substances increased the expression of the genes related to carbohydrate metabolism, including glycolysis in various species such as maize, poplar and Arabidopsis, respectively. SnRK1, a central regulator of cellular energy homeostasis, was upregulated, which may lead to the regulation of gene expression associated with carbohydrate metabolism in compost-treated plants, thereby resulting in the control of multiple developmental processes including root cell growth and proliferation [42]. On the other hand, the expression of some key enzymes in the glycolysis pathway, such as GAPDH, FBPA, 1-SST and AcsA, which could be used to produce an array of different types of carbohydrates, was found to be decreased. Moreover, thiamine pyrophosphate carrier (TPC) involved in thiamine pyrophosphate (TPP) across the mitochondrial membrane was downregulated. TPP is the active form of thiamine (Vitamin B) and it serves as a cofactor for several enzymes involved in energy metabolism, including pyruvate dehydrogenase, a key enzyme in the Krebs cycle, the α-ketoglutarate dehydrogenase complex, and cytosolic transketolase, all of which are involved in carbohydrate catabolism [43]. Other metabolic changes associated with energy production observed under compost treatment include a decrease in the levels of ATP synthase, which converts adenosine diphosphate (ADP) and phosphate to adenosine triphosphate (ATP) from the electron transport-generated proton gradient. It is well known that under stressed conditions, carbohydrates can act as osmoprotectants that remove reactive oxygen species (ROS) and stabilise cellular membranes, which can protect plants from oxidative damage [44]. Under stressed conditions, several previous studies, such as those on poplar [41], Arabidopsis [23], maize [45] and potato [46], have shown that the application of biostimulants and organic amendments increases the expression of various proteins that often participate in energy and carbohydrate metabolism. These results suggest that enzymes associated with the glycolytic pathway might assist in the intensification of glycolysis to provide energy for supporting plant growth and metabolic activities under abiotic and biotic stresses. In this study, under unstressed conditions, the compost treatment decreased the most identified protein related to the glycolysis pathway, indicating that photosynthesis may be the main process of energy source for plant cells for the promotion of plant growth and development in barley plants. However, under stress, photosynthesis is blocked, and glycolysis and the TCA cycle will provide energy for the growth and the development of plants [47]. 

### 3.7. Amino Acids Metabolism Proteins

Amino acids play key roles in several metabolic processes of plants such as plant nutrition. The application of the compost induced differential changes in the enzymes related to amino acid metabolism, such as delta-1-pyrroline-5-carboxylate synthase (P5CS), asparagine synthetase (ASNS), arginine decarboxylase (ADC) and O-phosphoserine phosphohydrolase (PSP). Proteins encoding P5CS and ASNS involved in proline (Pro) and asparagine (Asn) synthesis, respectively [48], showed higher abundance levels in the barley roots under the compost treatment. These increased amino acids may be involved in physiological processes associated with plant growth enhancement such as protein biosynthesis. Pro is known to play a vital role in regulating general protein synthesis in plants. Pro is involved in a wide range of functions in plant growth and development such as cell wall elongation and modifications because it is a key determinant of many cell wall proteins, root and shoot growth, inflorescence architecture, embryo formation/seed development and seed germination [49]. Moreover, these authors noted that Pro also maintains and protects proteins required for cell differentiation and division during stress conditions. Asn is also involved in a wide range of functions in plant growth and development due to its critical role in nitrogen storage and transport between plant cells and organs in many plant species [50]. An increase in some amino acid content, paired with a greater abundance of ribosomal proteins such as 60S and a decreased abundance of carboxypeptidase (CP) and serine carboxypeptidase-like 51 (SCPL) responsible for the breakdown of proteins [51] may result in enhanced protein synthesis, accounting for the plant root responses which lead to the higher biomass production. Ribosomal proteins (RPs) are not only essential for protein production, but they also play an important role in cell division, growth, proliferation and differentiation, thereby promoting plant growth and development [52]. Enhanced protein synthesis in biostimulant-treated plant roots such as HS has been previously demonstrated in Arabidopsis roots [23], poplar roots [41] and maize roots [45]. On the other hand, an increase in the abundance of ADC, which catalyses the conversion of arginine (Arg) into agmatine and carbon dioxide [53] and decreases the abundance of PSP, which is involved in both serine (Ser) and glycine (Gly) biosynthesis [54], may result in a decrease in the levels of Arg, Ser and Gly in the roots of compost-treated plants. These results suggest possible active utilisation of these metabolites in the cellular milieu. Interestingly, the decreased levels of certain amino acids could be related to their active involvement in energy metabolism [45]. Indeed, Ser and Gly can be degraded into pyruvate, which is the primary respiratory substrate for energy production to support plant growth and development. However, Arg can be recycled into ammonia, an appropriate nitrogen source required for growth improvement [55]. Moreover, Arg can be degraded into ornithine to generate putrescine, a polyamine that may play a crucial role in many physiological processes in plants, such as cell division, root formation, root growth and initiation of lateral root formation [56,57], thereby improving plant growth and development. 

### 3.8. Folding, Synthesis and Degradation Proteins

The balance between protein synthesis and protein degradation controls plant growth rate and development as well as stress responses [58]. In this study, the compost treatment decreased the abundance of elongation factors (EF1 and EF3) that play a central role in the protein biosynthesis on the ribosome [59], as well as the small ribosomal subunit (40S) that participates in scanning for messenger RNAs and initiation of protein synthesis; however, it increased the abundance of 26S proteasome non-ATPase regulatory subunit 6, which is involved in numerous cellular processes, including cell cycle progression, indicating a negative impact of the compost treatment on protein synthesis. This may be due to the fact that, when fertilized with compost, barley-treated plants were inclined to distribute more energy and substances for metabolic processes other than protein synthesis, since ribosome biosynthesis is a process with high energy demand [60]. This will result in limiting excessive energy input into the ribosome pathway, thereby resulting in more energy, which could be invested in promoting plant growth and development [41]. Previous studies [23,61] also found that HS can stimulate proteasome complex-related enzymes, such as 26S proteasome non-ATPase regulatory subunit 14 and 26S proteasome non-ATPase regulatory subunit 6 in Arabidopsis and maize roots, respectively. Moreover, the increased abundance of 26S suggests that proteasome complex, a widely used mechanism of compost-treated cells to control protein activities, is often involved in the regulation of numerous plant signalling and metabolic pathways. This is coherent with the view that the proteolytic capacity of a cell is expected to be the result of a careful balancing act that reflects environmental conditions and developmental stage [62].

Protein folding-related proteins were also detected in the barley roots treated with compost; these included proteins such as endoplasmin, chaperonin CPN60-like, heat shock protein (HSP 70), and small heat shock protein (sHSP). These results were consistent with previous investigations [33,61], who reported that HS affects the abundance of proteins related to protein folding in roots of Arabidopsis [33] and maize, respectively. Plant heat shock proteins (HSPs), as chaperones, play a critical role during the normal life cycle of plants as well as a pivotal role in conferring biotic and abiotic stress tolerance. These proteins participate in protein quality control and guarantee protein functionality under control and stress conditions because they prevent protein misfolding or aggregation. Indeed, their function in plants is crucial for normal growth, development and response to diverse stresses [63]. In this study, endoplasmin and chaperonin CPN60-like were upregulated in the barley root-treated plants; however, heat shock protein (HSP 70) and small heat shock protein (sHSP) were downregulated. These results were consistent with those reported previously [64]. These authors noted that biostimulants based on *Ascophyllum nodosum* extracts (named Phylgreen) and based on animal L-amino acids (named Delfan Plus) induced the expression of some HSPs under control conditions, suggesting that they play a role during plant development, while other HSPs such as HSP 70 and small heat shock protein (sHSP) were upregulated under heat stress, suggesting that they perform a protective function against abiotic stress including heat stress.

### 3.9. Regulation Processes Proteins

Another category highlighted was proteins involved in regulation processes. Proteins involved in RNA processing, such as rRNA 2′-O-methyltransferase fibrillarin 1, were identified in compost-treated roots, where this enzyme (which is involved in pre-rRNA processing by catalysing the site-specific 2′-hydroxyl methylation of ribose moieties in pre-ribosomal RNA [65]) was upregulated, indicating that compost can modify the protein synthesis pathway, influencing the barley root development. Protein modification plays a key role in many cellular functions, such as plant growth and development. Histones are also key determinants of gene regulation during development, since they are responsible for DNA condensation, organisation and regulation in the nucleus, impacting accessibility and effectiveness of the transcriptional machinery [66]. In this study, the level of histone H2B increased under the compost treatment; however, the level of histone H2A decreased. These results were consistent with those reported previously in the roots of maize [61] and wheat [67]. According to these authors, proteins involved in regulation processes were differentially represented in HS-treated maize plants and wheat plants treated with marine and fungal-based biostimulants.

### 3.10. Cytoskeleton Dynamics Proteins

Cytoskeleton proteins, such as tubulin (tubulin alpha-chain), were upregulated in the barley roots treated with compost. Similarly, previous results reported that an application of HS increased the abundance of the tubulin beta-chain [61]. Tubulin is a major component of microtubules that are cytoskeletal components involved in pivotal eukaryotic functions such as cell division and cell cycle, intracellular trafficking, and cell wall construction [68]. 

### 3.11. Plant Hormone Metabolism Proteins

In the present study, the compost treatment demonstrated that induced proteins that play critical roles in plant metabolism serve as precursors for a variety of plant hormones such as carboxylesterase (CXE), which may in turn act as precursors of endogenous plant hormones [69]. The authors note that CXE may be related to hormone regulating pathways since they contain cis-elements related to plant hormones gibberellin (GA) and indole acetic acid (IAA). Indeed, IAA is the most common plant hormone of the auxin class and it regulates various aspects of plant growth and development including lateral root formation and elongation [70]. In this context, GA stimulates polar auxin transport and shares a common transcriptome with auxin, including many transcripts related to cell elongation and expansion during plant growth and development [71].

### 3.12. Secondary Metabolism Proteins

Plant secondary metabolites perform a variety of functions in plant growth and developmental processes. The phenylpropanoids and flavonoids biosynthesis are mainly involved in the biosynthesis of secondary metabolites [72]. Trans-cinnamate 4-monooxygenase (TCM) was upregulated under the compost treatment; this confirms that phenylpropanoids biosynthesis and flavonoids biosynthesis were indeed impacted by the compost treatment. In support of our postulation, previous studies have shown that proteins related to the phenylpropanoid pathway were found to significantly increase following the application of biostimulants such as HS and microbial-based biostimulants [23,73]. Although their presence is usually related to plant defense, as well as being part of the antioxidant systems of plants, the accumulation of phenols and flavonoids influences the plant growth promotion process. In this context, these compounds could be described as fundamental as signal molecules in the barley roots cell wall biogenesis under the compost treatment.

Lignin is a phenolic cell wall polymer and is one of the important products of the plant phenylpropanoid biosynthesis pathway that play an important role in the development of plants since they provide a structural framework to support plant growth. Proteomic analysis showed that the expression of caffeic acid methyltransferase (COMT), which is one of the key genes regulating lignin synthesis [74], was increased in the barley roots under the compost treatment, suggesting that the compost application may increase the lignin content of cell wall biomass, which could lead to accelerated root growth rates, tolerance of environmental stresses, as well as resistance to pathogen infection.

## 4. Materials and Methods

### 4.1. Plant Growth and Treatment with Date Palm Waste Compost

A field study was conducted from December 2020 to June 2021 at the production station of ASOC (Association for Saving Oasis of Chenini, Gabes, Tunisia). The soil texture was sandy loam. The acidity (pH) of the soil was 7.50, soil organic matter was 0.90% and total organic carbon content was 0.52%. Moreover, the soil used in this study had total N, available P and exchange K content of 0.28 g kg^−1^, 4.92 mg kg^−1^ and 292 mg kg^−1^, respectively. The area has a Mediterranean arid climate, with an annual mean precipitation of 160.20 mm and an annual mean temperature of 20.06 °C. Three 2.00 m × 3.00 m plots were established for each of the two treatments (a total of 6 plots): control and soil amended with 30 t ha^−1^ of date palm waste compost. Physico-chemical and biological analyses of the date palm waste compost used in this study are presented in Table 3. The seeds of the local barley (*Hordeum vulgare* L.) cultivar, Sahli, were obtained from the Technical Centre of Organic Agriculture (TCOA, Gabes, Tunisia) and were sown using a seed rate of 120 kg ha^−1^. The crop was maintained in the field using standard agronomic practices. Various morpho-agronomical parameters were recorded during the tillering stage of the plants. Grain yield and its attributes were recorded at maturity. Root tissue harvested from control and compost-treated plants at the tillering stage were treated with liquid nitrogen and then stored at −80 °C for further molecular analysis.

### 4.2. Extraction, Digestion and LC-MS/MS Analyses

Total proteins from the root samples (100 mg) were extracted by phenol method [75]. Frozen powder was transferred to a 50 mL centrifuge tube, before 10 mL of lysis buffer (containing 0.5MTris-HCl pH 7.5, 0.7 M sucrose, 50 mM EDTA, 0.1 M KCl, 10 mM thiourea, 2 mM phenylmethylsulfonyl fluoride and 2% 2-mercaptoethanol) was added. A similar amount of water-saturated phenol was added to the sample, before the sample was incubated for 30 min on a shaker at 4 °C. After centrifugation for 30 min at 12,000 rpm and at 4 °C, the phenol phase was recovered and proteins were precipitated by adding the equivalent of five volumes of 0.1 M ammonium acetate/methanol and incubating overnight at −20 °C. Finally, the protein precipitates were washed with methanol and then with cold acetone, and dissolved in RUT Buffer (6 M urea, 2 M thiourea, 10 mM DDT, 30 mM Tris HCl pH 8.8, RapiGest 0.1%). The protein concentration was measured using a Plusone 2D Quant kit (GE Healthcare) according to the manufacturer’s instructions. Proteins were incubated for 30 min and then alkylated with 50 mM iodoacetamide for 60 min in the dark and at room temperature. The protein samples were then diluted ten-fold in 50 mM ammonium bicarbonate buffer and digested overnight with 800 ng trypsin at 37 °C. The digested peptides were vacuum dried using a polymeric C18 column (Phenomenex, Torrance, CA, USA) according to the manufacturer’s instructions. 

Liquid chromatography-tandem mass spectrometry (LC-MS/MS) analyses were performed on a NanoLC-Ultra System (nano2DUltra, Eksigent, Les Ulis, France) connected to a Q Exactive PLUS mass spectrometer (Thermo Electron, Waltham, MA, USA) on the PAPPSO proteome analysis platform (http://pappso.inra.fr) [76]. For each sample, approximately 400 ng of protein digest were loaded onto a Biosphere C18 precolumn (0.1 × 20 mm, 100 Å, 5 µm; nanoseparation) at 7.5 µL min^−1^ and desalted with 0.1% formic acid and 2% acetonitrile. After 3 min, the pre-column was connected to a Biosphere C18 nanocolumn (0.075 × 300 mm, 100 Å, 3 µm; nanoseparation). The enriched peptides were dissolved in mobile phase A containing 0.1% formic acid in water. The elution gradient was increased from 5% to 35% in mobile phase B (0.1 formic acid in 100% acetonitrile) for 110 min. One run lasted 120 min, including the regeneration step at 95% buffer B and the equilibration step at 100% buffer A.

In order to obtain low mass range data suitable for protein quantification, an MS scan was performed with full scans at a 75,000 resolution and MS/MS scans at a 17,500 resolution. MS/MS was repeated for the eight most intense ions detected in full scan and dynamic exclusion was set to 40 s [77].

### 4.3. Identification and Quantification of Proteins 

Protein identification was performed using the protein sequence database of *Hordeum vulgare* downloaded from UniProt database (https://www.uniprot.org/, accessed 1 August 2022), with 58,482 entries. Standard contaminant sequences were interrogated as well as the decoy database which included *Hordeum vulgare* inverse sequences. Database search was performed with X!Tandem (v2015.04.01.1; http://www.thegpm.org/TANDEM/, accessed on 26 November 2021). Identified proteins were filtered and sorted by using X!TandemPipeline [78]. Enzyme specificity was set to trypsin with one and five possible missed cleavages in the first and refined pass, respectively. Only proteins identified with at least two independent, unique peptides per protein with an eValue smaller than 0.01 were considered [79]. For protein inference, only the proteins identified with a minimum of two peptides were considered as valid. Protein inference was performed using all samples together. Based on extracted ion chromatograms (XICs), peptide ions were quantified using the software MassChroQ version 2.4.8. Relative protein abundance was calculated with the “model” method based on peptide intensity modelling [80]. A fold change of treatment versus control of ≥1.50 was set as the threshold for increased abundance; while a fold change of ≤0.66 (1/1.50) was considered to indicate decreased protein content (Table 2).

### 4.4. Bioinformatics Analysis

Functional annotations of differentially abundant proteins (DAPs) were performed using the UniProt-GOA database (http://www.ebi.ac.uk/GOA/) and the PANTHER Classification System (www.pantherdb.org). DAPs were classified according to four categories: biological process, molecular function, protein class and cellular component. The sequence analysis application InterProScan was performed to obtain a functional description of identified protein domain based on the protein sequence alignment method, using the InterPro domain database. The Kyoto Encyclopedia of Genes and Genomes (KEGG: https://www.genome.jp/kegg/) database was used to identify enriched pathways [81]. 

To construct a protein–protein interaction (PPI) network for the identified DAPs, the Search Tool for the Retrieval of Interacting Genes/Proteins (STRING) web-based program (version 11.5) (http://www.string-db.org/ accessed on 1 August 2022) was used [82]. For PPI, a database for *Hordeum vulgare* was searched with highest confidence (0.70).

### 4.5. Quantitative Real-Time PCR (qRT-PCR)

Total RNA from barley root tissue was extracted according to Chang et al. [83]. cDNA was synthesised from 1 μg of the total RNA using a revert Aid First Stand cDNA Synthesis Kit (Biomatik; Wilmington, DE, USA) according to the manufacturer’s instructions. Primer3 Input (version 0.4.0) software [84] (http://frodo.wi.mit.edu/primer3/) was used to design gene specific primers (Table 4). The housekeeping gene actin of barley was used as the internal control. The qRT-PCR was performed on a 7300 Real-Time PCR Detection System (Applied Biosystems, Foster City, CA, USA) in a 30 μL volume containing 15 μL (2X) Maxima SYBR Green/ROX qPCR Master Mix (Biomatik; Wilmington, DE, USA), 2 µL cDNA template (50–100 ng), 1 µL (10 mM) of forward and reverse primers and 12 µL double-distilled H_2_O. PCR conditions were the following: 95 °C for 10 min, 40 cycles of 30 s at 95 °C, 60 °C for 1 min. The PCR was followed by a melt curve analysis by heating from 60 °C to 95 °C with increments of 0.5 °C/cycle. Relative gene expression was calculated according to the delta–delta Ct method [85]. Three biological replicates were performed for each experiment.

### 4.6. Statistical Analysis

All the results are presented as mean values ± standard deviation (SD) of at least three independent experiments. Data were subjected to one-way analysis of variance (ANOVA) using the SPSS 23.0 statistical program (SPSS Science, Chicago, IL, USA) and the means were compared using Tukey’s Honestly Significant Difference (HSD) test (*p* < 0.05).

## 5. Conclusions

**Figure 6:**
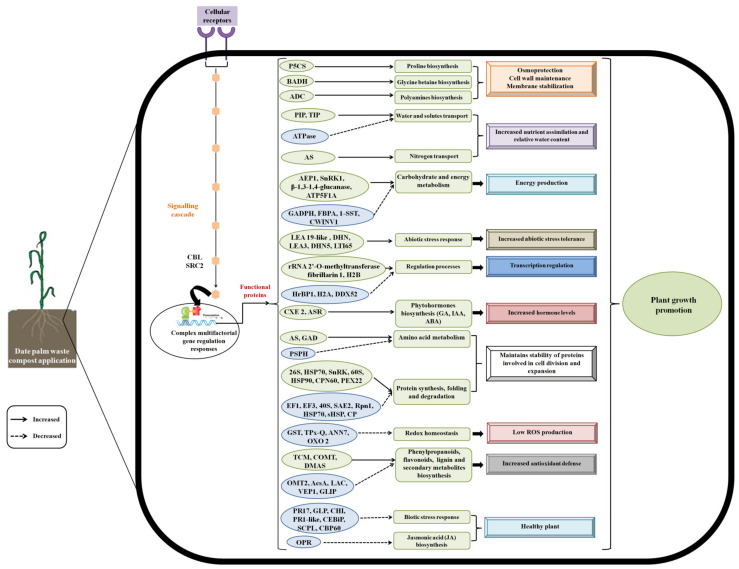
A model of date palm waste compost effects on barley plants.

The date palm waste compost tested in this study showed positive effects on the barley plants, as it stimulated plant growth. The variation in protein abundances of the barley roots in response to the compost treatment was investigated. A large number of DAPs in the barley roots fertilized with the compost was identified, suggesting that the regulation effect of the compost on the barley involves multiple metabolism processes. Proteomic analysis indicated that the compost treatment altered the proteins involved in glycolysis and energy metabolism, protein synthesis and degradation, RNA processing, stress response, biosynthesis of amino acids, the phenylpropanoid pathway, and plant hormone signalling transduction (Figure 6). Future investigations are necessary to verify further these expression patterns revealed by the proteomic analysis. Results obtained from this study indicate that the activation of pathways involved in the production of primary and secondary metabolites are essential for barley plant growth, providing novel insights into the molecular mechanisms regulating the response to compost as organic fertilizer in plants.

## Figures and Tables

**Table 1:** Effect of date palm waste compost on growth and yield of barley plants. Different letters denote significant differences (Tukey’s HSD, *p* < 0.05).

Treatment	Shoot Length (cm)	Root Length (cm)	Shoot Dry Weight (g)	Root Dry Weight (g)	Grain Yield (t ha^−1^)
Control	32.00 ± 2.00 ^b^	14.83 ± 1.75 ^a^	2.78 ± 0.13 ^b^	1.18 ± 0.09 ^b^	2.89 ± 0.54 ^b^
Compost	49.66 ± 1.52 ^a^	16.90 ± 2.12 ^a^	5.99 ± 0.25 ^a^	1.44 ± 0.07 ^a^	4.33 ± 0.67 ^a^

**Table 2:** Differentially abundant proteins (DAPs) in roots of barley under date palm waste compost treatment.

No	Protein Name	Function	Regulated Type	Fold Change	Protein Accession	KEGG Pathway
1	Peroxisome biogenesis protein 22-like (PEX22)	Protein translocation	Up	1.50	F2DXG1	Peroxisome
2	Delta-1-pyrroline-5-carboxylate synthase (P5CS)	Amino acid metabolism	Up	1.51	A0A224MLP5	Biosynthesis of amino acids
3	60S ribosomal protein L7a (60S)	Protein biosynthesis	Up	1.51	F2DE13	Ribosome
4	Trans-cinnamate 4-monooxygenase (TCM)	Flavonoid biosynthesis	Up	1.54	F2E6M8	Biosynthesis of secondary metabolites
5	Tonoplast intrinsic protein (TIP)	Solute transport	Up	1.55	D2KZ38	Transporters
6	Dehydrin	Stress response	Up	1.56	M0UW32	Protein processing
7	Betaine aldehyde dehydrogenase (BADH)	Glycine betaine biosynthesis	Up	1.59	Q94IC0	Metabolic pathways
8	Caffeic acid 3-O-methyltransferase (COMT)	Lignin biosynthesis	Up	1.60	F2D692	Biosynthesis of secondary metabolites
9	Aldose 1-epimerase (AEP 1)	Galactose metabolism	Up	1.61	M0X1Y4	Glycolysis/gluconeogenesis
10	Deoxymugineic acid synthase 1 (DMAS)	Phytosiderophore biosynthesis	Up	1.61	F2DHN0	Biosynthesis of secondary metabolites
11	Calcium-binding protein CBP-like (CBL)	Multi-process regulation	Up	1.64	A0A287NLZ0	Plant-pathogen interaction
12	Annexin D7 (ANN7)	Stress response	Up	1.64	A0A287UPG1	Calcium signaling pathway
13	La protein 1-like	RNA biosynthesis transcription	Up	1.66	F2DIB4	Transcription
14	Low-temperature-induced 65 kDa protein-like isoform X1 (LTI65)	Stress response	Up	1.67	F2D3S4	Plant hormone signal
15	PIP aquaporin isoform (PIP)	Solute transport	Up	1.70	A0A287IVR7	Transporters
16	rRNA 2′-O-methyltransferase fibrillarin 1	Protein biosynthesis	Up	1.71	F2E7G3	Ribosome
17	Late embryogenesis abundant protein 19-like (LEA 19-like)	Stress response	Up	1.72	F2CUJ9	Protein processing
18	Carboxylesterase 2 (CXE 2)	Hydrolysis	Up	1.79	A0A287TVH9	Plant hormone signal
19	Protein SRC2 homolog (SRC2)	Signal transduction	Up	1.80	F2EAD0	Calcium signaling pathway
20	Chaperonin CPN60-like 2 (CPN60)	Protein homeostasis	Up	1.80	F2CZD9	RNA degradation
21	Arginine decarboxylase (ADC)	Putrescine biosynthesis	Up	1.92	M0XCI1	Metabolic pathways
22	Asparagine synthetase (AS)	Amino acid metabolism	Up	2.06	A0A287P6Q4	Biosynthesis of amino acids
23	Endoplasmin (HSP90)	Protein homeostasis	Up	2.27	P36183	Protein processing
24	SNF1-related protein kinase regulatory subunit gamma-1 (SnRK)	Protein modification	Up	2.30	M0XDA4	MAPK signaling pathway
25	Abcissic acid stress and ripening (ASR)	Stress response	Up	2.41	A0A1Z3GD05	Plant hormone signal
26	Late embryogenesis abundant protein (LEA3)	Stress response	Up	2.44	B5TWC9	Protein processing
27	Glutamate decarboxylase (GAD)	Amino acid metabolism	Up	2.89	F2E7E5	Biosynthesis of secondary metabolites
28	Embryonic protein DC-8-like isoform X1	Embryogenesis	Up	3.26	A0A287PP23	Protein processing
29	(1-3,1-4)-beta-D-glucanase	Hydrolysis	Up	3.33	Q02345	Metabolic pathways
30	Histone H2B	Chromatin organisation	Up	3.72	A0A287T8I9	Necroptosis
31	Tubulin alpha chain	Cytoskeleton organisation	Up	4.09	F2E847	Metabolic pathways
32	Dehydrin (DHN5)	Stress response	Up	4.33	Q40042	Protein processing
33	26S proteasome non-ATPase regulatory subunit 6	Protein homeostasis	Up	5.59	F2DJW8	Proteasome
34	Glyceraldehyde-3-phosphate dehydrogenase (GAPDH)	Carbohydrate degradation	Down	0.25	F2D714	Glycolysis/gluconeogenesis
35	GDSL esterase/lipase (GLIP)	Hydrolysis	Down	0.28	A0A287TDE4	Biosynthesis of secondary metabolites
36	Oxalate oxidase 2 (OXO 2)	H_2_O_2_ generation	Down	0.30	P45851	Glyoxylate and dicarboxylate metabolism
37	SUMO-activating enzyme subunit 2 (SAE2)	Protein homeostasis	Down	0.34	A0A287HPI7	Ubiquitin mediated proteolysis
38	Harpin binding protein 1 (HrBP1)	RNA-binding protein	Down	0.35	Q5QJB5	Protein processing
39	Fructose-bisphosphate aldolase (FBPA)	Gluconeogenesis	Down	0.37	F2ELD1	Glycolysis/gluconeogenesis
40	Dolichyl-diphosphooligosaccharide--protein glycosyltransferase subunit 1 (Rpn1)	Protein modification	Down	0.38	M0Z968	Metabolic pathways
41	Plasma membrane ATPase	Solute transport	Down	0.39	F2DW68	Calcium signaling pathway
42	Elongation factor 1-alpha (EF1)	Protein biosynthesis	Down	0.40	F2D525	AMPK signaling pathway
43	Calmodulin-binding protein 60 B-like isoform X2 (CBP60)	Stress response	Down	0.41	F2DIS1	Calcium signaling pathway
44	Pathogenesis related protein (PR17)	Plant defense responses	Down	0.44	A7YA66	MAPK signaling pathway
45	40S ribosomal protein SA (40S)	Protein biosynthesis	Down	0.45	F2D7E4	Ribosome
46	Serine carboxypeptidase-like 51 (SCPL)	Hydrolysis	Down	0.45	A0A287WND1	Biosynthesis of secondary metabolites
47	Pathogenesis-related protein 1-like (PR1-like)	Plant defense responses	Down	0.46	A0A287VZQ8	MAPK signaling pathway
48	O-methyltransferase 2 (OMT2)	Aromatic compound biosynthetic process	Down	0.47	A0A287PZJ8	Metabolic pathways
49	O-phosphoserine phosphohydrolase (PSPH)	Amino acid metabolism	Down	0.47	F2D8E1	Biosynthesis of amino acids
50	Lipase	Lipid hydrolysis	Down	0.47	UCW116	Metabolic pathways
51	Histone H2A	Chromatin organisation	Down	0.49	F2DGG6	Necroptosis
52	Glutathione S-transferase (GST)	Redox homeostasis	Down	0.49	F2D4L0	Glutathione metabolism
53	Chitinase (CHI)	Plant defense responses	Down	0.50	F2CQW5	Amino sugar and nucleotide sugar metabolism
54	Acetyl-coenzyme A synthetase (AcsA)	Fatty acid production	Down	0.51	F2DVB4	Glycolysis/gluconeogenesis
55	Germin-like protein 2a (GLP)	Plant defense responses	Down	0.51	Q0GR10	Glyoxylate and dicarboxylate metabolism
56	Thioredoxin-dependent peroxiredoxin (TPx-Q)	Redox homeostasis	Down	0.53	F2DTT4	Peroxisome
57	DEAD-box ATP-dependent RNA helicase 52C (DDX52)	Vesicle trafficking	Down	0.54	A0A287NPY4	RNA degradation
58	Leucine-rich repeat extensin-like protein 6	Cell wall organisation	Down	0.56	A0A287TFV1	Metabolic pathways
59	Heat shock protein (HSP 70)	Protein homeostasis	Down	0.56	F2DT51	MAPK signaling pathway
60	Thiamine pyrophosphate carrier 1	Solute transport	Down	0.56	F2DJG4	Transporters
61	Chitin elicitor-binding protein (CEBiP)	Stress response	Down	0.57	F2D6S9	Plant-pathogen interaction
62	Elongation factor 3 (EF3)	Protein biosynthesis	Down	0.58	F2DTZ6	Protein processing
63	Sucrose 1-fructosyltransferase (1-SST)	Fructan biosynthesis	Down	0.61	J7GM45	Starch and sucrose metabolism
64	12-oxophytodienoic acid reductase (OPR)	Oxylipins biosynthesis	Down	0.62	M1EUV0	Biosynthesis of secondary metabolites
65	3-oxo-Delta(4,5)-steroid 5-beta-reductase (VEP1)	Enzyme oxidoreductase	Down	0.62	M0Y0N6	Metabolic pathways
66	ATP synthase subunit alpha (ATP5F1A)	Cellular respiration	Down	0.62	F2DBC9	Metabolic pathways
67	Multiprotein-bridging factor 1a-like	Transcription regulation	Down	0.63	F2CW93	Signal transduction
68	Beta-fructofuranosidase, insoluble isoenzyme 4-like isoform X1 (CWINV1)	Hydrolysis	Down	0.63	A0A287T167	Biosynthesis of secondary metabolites
69	Laccase (LAC)	Enzyme oxidoreductase	Down	0.64	F2DUK8	Metabolic pathways
70	Carboxypeptidase (CP)	Hydrolysis	Down	0.65	F2DJ52	Protein digestion and absorption
71	Small heat shock protein (sHSP)	Protein homeostasis	Down	0.65	A0A287RG57	Protein processing
72	Plant SNARE 13 (PSN13)	Vesicle trafficking	Down	0.66	M0X338	SNARE interactions in vesicular transport

**Table 3:** Physico-chemical properties of the experimental soil.

Parameters	Value
Total organic carbon (%)	18.58
Total N (%)	1.21
C/N	15.36
P (%)	0.54
K (%)	0.95
Ca (%)	8.18
Mg (%)	1.05
Na (%)	0.42
Alkalinity (% CaCO3)	11.50
Zn (mg kg^−1^ DW compost)	70.10
Fe (g kg^−1^ DW compost)	70
Mn (mg kg^−1^ DW compost)	130
Cu (mg kg^−1^ DW compost)	11.60
Cd (mg kg^−1^ DW compost)	0.20
Pb (mg kg^−1^ DW compost)	4.15
Cr (mg kg^−1^ DW compost)	11.50
Ni (mg kg^−1^ DW compost)	5.88
Total coliforms (MPN g DW^−1^ compost)	143.33 ± 5.77
Faecal coliforms (MPN g DW^−1^ compost)	120 ± 17.32
*Escherichia coli* (MPN g DW^−1^ compost)	114 ± 23.79
Faecal Streptococci (MPN g DW^−1^ compost)	114.33 ± 23.8
*Salmonella* spp. (MPN g DW^−1^ compost)	<0.3
*Shigella* spp. (MPN g DW^−1^ compost)	<0.3

**Table 4:** Sequences of primers used for real time quantitative PCR (qRT-PCR).

Gene	Sequence (5′–3′)	Product Size (bp)	Tm (°C)
Plasma membrane ATPase (*HvATPase*)	F: 5′-CGTTGGTGTCTCCATTGTTG-3′ R: 5′-TTGCAACCGGTGGTGTAGTA-3′	105	60
Pathogenesis related protein PR17 (*HvPR17*)	F: 5′-CAGAGCTGATGGTCGACGTA-3′ R: 5′-CGCTCCAGTCAATACAGCAA-3′	122	60
Glutathione S-transferase (*HvGST*)	F: 5′-AAGCTGTACGGGATGATGCT-3′ R: 5′-GGTTGAGCTTGAGGAAGTCG-3′	139	60
Chitinase (*HvCHI*)	F: 5′-CTACACGTACGACGCCTTCA-3′ R: 5′-TAGTCTCGTGGGAGGTCTGG-3′	122	61
Thioredoxin-dependent peroxiredoxin (*HvTPx-Q*)	F: 5′-GTCCAAGAAAACCCAGACGA-3′ R: 5′-AAACGGCCATGACAAAACTC-3′	149	60
Heat shock protein (*HvHSP*)	F: 5′-CTCAAATCGAGATCCCCGTA-3′ R: 5′-TCGATCTTGGCTTGTCCTCT-3′	118	60
Tonoplast intrinsic protein (*HvTIP*)	F: 5′- GCTTCCTCCTCCGCTTCT-3′ R: 5′- ATGACGATCTCCAGGACCAC-3′	97	59
Dehydrin (*HvDHN*)	F: 5′-CGTGTCAAGATGGAGGGATT-3′ R: 5′-CTGAAGCCCGTATACCCAAA-3′	98	60
Aquaporin (*HvPIP*)	F: 5′-CTGGCCACTATCCCAATCAC-3′ R: 5′-CACCCAGAAGATCCAGTGGT-3′	111	60
Late embryogenesis abundant protein 19-like (*HvLEA19-like*)	F: 5′-ACGAAGGAGAGGGACAGGAT-3′ R: 5′-CGCGTACAGATTTCCAGACA-3′	106	59
Late embryogenesis abundant protein LEA3 (*HvLEA3*)	F: 5′-CATGGGAGGGGACAACAC-3′ R: 5′-GATTCCTGGTGGTGGTGGT-3′	86	61
Dehydrin DHN5 (*HvDHN5*)	F: 5′-TTACATGCCGACACTTCCAA-3′ R: 5′-CGAAAACATCCGATCCTTGT-3′	85	59
Actin (*HvActin*)	F: 5′-CGACAATGGAACCGGAATG-3′ R: 5′-CCCTTGGCGCATCATCTC-3′	56	61

## Data Availability

Data is contained within the article and supplementary material.

## Supplementary Materials

The following supporting information can be downloaded at: https://www.mdpi.com/article/10.3390/plants12030526/s1, Figure S1: correlation coefficient (R^2^); Table S1: The total identified proteins.
